# Fourteen-day vonoprazan-based bismuth quadruple therapy for *H. pylori* eradication in an area with high clarithromycin and levofloxacin resistance: a prospective randomized study (VQ-HP trial)

**DOI:** 10.1038/s41598-024-59621-3

**Published:** 2024-04-18

**Authors:** Nuttapat Tungtrongchitr, Phubordee Bongkotvirawan, Sarita Ratana-Amornpin, Sith Siramolpiwat, Thanee Eiamsitrakoon, Pornpen Gamnarai, Arti Wongcha-um, Yoshio Yamaoka, Kammal Kumar Pawa, Ratha-korn Vilaichone

**Affiliations:** 1 Ramathibodi Medical School, Chakri Naruebodindra Medical Institute, Faculty of Medicine Ramathibodi Hospital, Mahidol University, Samut Prakan, 10540 Thailand; 2 Department of Medicine, Faculty of Medicine, Center of Excellence in Digestive Diseases and Gastroenterology Unit, Thammasat University, Pathum Thani, 12120 Thailand; 3 Chulabhorn International College of Medicine (CICM), Thammasat University, Pathum Thani, 12120 Thailand; 4 Department of Environmental and Preventive Medicine, Faculty of Medicine, Oita University, Yufu, Oita 879-5593 Japan; 5 Department of Medicine-Gastroenterology, Baylor College of Medicine, Houston, TX 77030 USA

**Keywords:** Gastric cancer, Gastritis

## Abstract

Potassium-competitive acid blockers (P-CABs) provide potent acid inhibition, yet studies on P-CAB-based quadruple therapy for *H. pylori* eradication are limited. We theorized that integrating bismuth subsalicylate into a quadruple therapy regimen could enhance eradication rates. However, data on the efficacy of vonoprazan bismuth quadruple therapy are notably scarce. Therefore, the aim of this study was to evaluate the efficacy of vonoprazan-based bismuth quadruple therapy in areas with high clarithromycin and levofloxacin resistance. This was a prospective, single-center, randomized trial conducted to compare the efficacy of 7-day and 14-day vonoprazan-based bismuth quadruple therapy for *H. pylori* eradication between June 1, 2021, and March 31, 2022. Qualified patients were randomly assigned to the 7-day or 14-day regimen (1:1 ratio by computer-generated randomized list as follows: 51 patients for the 7-day regimen and 50 patients for the 14-day regimen). The regimens consisted of vonoprazan (20 mg) twice daily, bismuth subsalicylate (1024 mg) twice daily, metronidazole (400 mg) three times daily, and tetracycline (500 mg) four times daily. *CYP3A4/*5 genotyping and antibiotic susceptibility tests were also performed. Successful eradication was defined as ^13^negative C-UBTs 4 weeks after treatment. The primary endpoint was to compare the efficacy of 7-day and 14-day regimens as first-line treatments, which were assessed by intention-to-treat (ITT) and per-protocol (PP) analyses. The secondary endpoints included adverse effects. A total of 337 dyspeptic patients who underwent gastroscopy were included; 105 patients (31.1%) were diagnosed with *H. pylori* infection, and 101 patients were randomly assigned to each regimen. No dropouts were detected. The antibiotic resistance rate was 33.3% for clarithromycin, 29.4% for metronidazole, and 27.7% for levofloxacin. The CYP3A4 genotype was associated with 100% rapid metabolism. The *H. pylori* eradication rates for the 7-day and 14-day regimens were 84.4%, 95% CI 74.3–94.2 and 94%, 95% CI 87.4–100, respectively (RR difference 0.25, 95% CI 0.03–0.53, p value = 0.11). Interestingly, the 14-day regimen led to 100% eradication in the clarithromycin-resistant group. Among the patients in the 7-day regimen group, only two exhibited resistance to clarithromycin; unfortunately, neither of them achieved a cure from *H. pylori* infection. The incidence of adverse events was similar in both treatment groups, occurring in 29.4% (15/51) and 28% (14/50) of patients in the 7-day and 14-day regimens, respectively. No serious adverse reactions were reported. In conclusion, 14 days of vonoprazan-based bismuth quadruple therapy is highly effective for *H. pylori* eradication in areas with high levels of dual clarithromycin and levofloxacin resistance.

## Introduction

*Helicobacter pylori* (*H. pylori*) is a gram-negative bacillus that colonizes the stomach. This bacterial colonization is strongly associated with lymphoid tissue (MALT) lymphoma, and gastric cancer^1–3^. Thus, *H. pylori* eradication is crucial for reducing the risk of gastric cancer. In recent years, several studies have reported a progressive decline in the effectiveness of conventional triple therapy for *H. pylori* eradication, with success rates decreasing to less than 80%^4,5^. According to the Bangkok consensus on *H. pylori* management in ASEAN, an alternative first-line regimen for *H. pylori* eradication is 10 days of sequential therapy or concomitant therapy. However, patients may have difficulty adhering to these regimens due to the complexity of their daily prescriptions, which frequently involve multiple tablets, resulting in poor adherence and an increased risk of adverse drug reactions^4,6^. The Maastricht VI/Florence consensus report suggested 14 days of bismuth-containing quadruple therapy as the first-line treatment. However, there is limited evidence regarding the efficacy of this regimen for eradicating *H. pylori* in Southeast Asia^7^.

The stability of gastric acid suppression is an essential factor affecting the success of *H. pylori* eradication. Vonoprazan, a recently approved potassium-competitive acid blocker (P-CAB) in Japan and the USA, has shown promising results in managing acid-related disorders, including *H. pylori* eradication. Due to its potent and prolonged suppression of gastric acid secretion, vonoprazan has been recommended for the treatment of erosive esophagitis, peptic ulcers, and *H. pylori* infection^7–10^.

Unlike proton pump inhibitors, vonoprazan is metabolized by *CYP3A4/5*, which may be advantageous for the rapid metabolism of the *CYP2C19* rapid metabolizer group. The *CYP2C19* genotype is a crucial predictor of successful *H. pylori* eradication in patients receiving omeprazole- or lansoprazole-based therapies^11,12^. A recent study confirmed the efficacy and safety of vonoprazan-based bismuth quadruple therapy for *H. pylori* eradication; however, information regarding the duration and efficacy of these regimens is still limited^13^.

We hypothesized that incorporating bismuth subsalicylate into a quadruple therapy regimen could improve eradication rates. Nonetheless, it is important to highlight the limited availability of data regarding the efficacy of vonoprazan bismuth quadruple therapy. The purpose of this study was to evaluate the effectiveness and tolerability of 7-day and 14-day vonoprazan-based bismuth quadruple therapy as a first-line *H. pylori* eradication protocol in Thailand. Antimicrobial susceptibility and *CYP3A4/5* genotyping were also tested.

## Materials and methods

### Study design and participants

This was a prospective, single-center, open-label randomized trial aimed at assessing the effectiveness and tolerability of vonoprazan-based bismuth quadruple therapy as a first-line treatment for *H. pylori* eradication in nonulcer dyspeptic patients. This study was conducted over a 10-month enrollment period at a tertiary care center in Thailand between June 1, 2021 and March 31, 2022. Patients aged between 18 and 70 years who underwent upper gastrointestinal (GI) endoscopy due to an indication as per the guidelines of the Gastroenterological Association of Thailand (GAT) and who were found to have *H. pylori* infection with nonulcer dyspeptic symptoms were enrolled in this study. The exclusion criteria were as follows: (1) history of upper gastrointestinal bleeding; (2) prior allergic reaction to amoxicillin, metronidazole, bismuth subsalicylate, or tetracycline; (3) use of antibiotics within one month prior to enrollment; (4) prior *H. pylori* eradication therapy; (5) current use of medication with potential drug interactions with the study medication; (6) presence of severe comorbidities such as end-stage renal disease, advanced cirrhosis, immunocompromised status, AIDS, malignancy, and/or cerebrovascular disease; (7) pregnancy or lactation; (8) history of arrhythmia or QTc prolongation; (9) previous surgical or endoscopic resection of the upper GI tract; (10) contraindication for gastric biopsy such as coagulopathy or current use of anticoagulants; and (11) unwillingness to participate in the study. This study was approved by The Human Research Ethics Committee of Thammasat University with approval number MTU-EC-IM-6-157/62 and was conducted according to good clinical practice guidelines and the Declaration of Helsinki. Written informed consent was obtained before enrollment. There were no changes in the protocol after trial commencement. This study has a data monitoring committee. This trial is registered with Thaiclinicaltrials.org, TCTR20230428003. Date of first registration: 28/04/2023.

### Interventions

**Figure 1:**
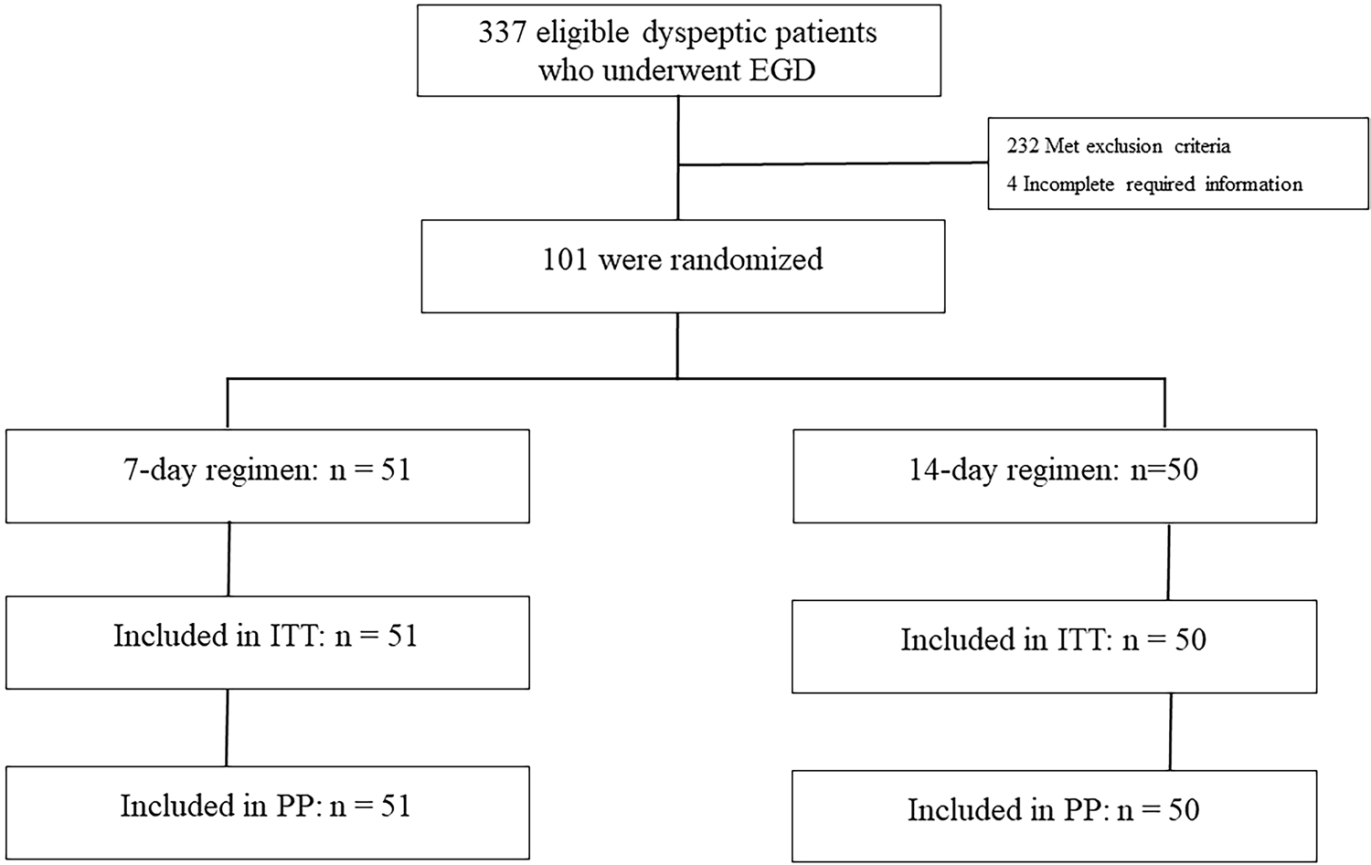
CONSORT flow diagram of this study.

The vonoprazan-based bismuth quadruple therapy consisted of twice-daily administration of vonoprazan (20 mg), bismuth subsalicylate (1024 mg), metronidazole (400 mg) 3 times daily and tetracycline (500 mg) 4 times daily for both the 7-day and 14-day regimens. Tetracycline was taken after meals and before bedtime. All medications were administered after meals. A flow diagram of this study is shown in Fig. 1. The 13C-urea breath test (UBT) was used to determine successful *H. pylori* eradication four weeks after the completion of the treatment period. To ensure proper adherence, patients were questioned about their medication intake, with nonadherence defined as taking less than 90% of the prescribed medication. Treatment-related adverse events were defined as any adverse event that resulted in death, hospitalization, disability, congenital anomaly, and/or required intervention to prevent permanent damage during the treatment period, while serious adverse events were defined as any medical occurrence resulting in disturbance of the patient’s daily life or requiring hospitalization. Furthermore, adverse events were assessed during follow-up at the outpatient clinic through questioning by physicians.

### Outcomes

This study aimed to compare the efficacy and tolerability of 7-day and 14-day vonoprazan-based bismuth quadruple therapy as the first-line treatment for *H. pylori* eradication in nonulcer dyspeptic patients. The primary endpoint was ^13^C-UBT, which was assessed by both intention-to-treat (ITT) and per-protocol analyses 4 weeks after therapy. The secondary endpoints included adverse effects, cost-effectiveness compared to previous regimens, and CYP3A4/5 genetic polymorphisms preventing *H. pylori* eradication in both groups. This study also evaluated the effectiveness of vonoprazan-based bismuth quadruple therapy against clarithromycin-resistant *H. pylori*, metronidazole-resistant, and dual-resistant strains. All secondary outcomes were analyzed using ITT analysis in participants with available data.

### Diagnosis of *H. pylori* infection and *CYP3A4* and *CYP3A5* genetic polymorphisms

Esophagogastroduodenoscopy was performed, and biopsies were collected from the gastric antrum and corpus for rapid urease testing, histological examination, *H. pylori* culture, and antibiotic susceptibility testing via the Epsilometer test (E-test) or GenoType®HelicoDR. Positivity for *H. pylori* infection was defined as (1) a positive *H. pylori* culture or (2) two positive tests (both rapid urease test and histology). Furthermore, blood samples were also collected from the participants for DNA extraction, which was subsequently used to perform *CYP3A4* and *CYP3A5* genotyping via the polymerase chain reaction-restriction fragment length polymorphism technique (New England Biolabs (NEB), Inc., USA). The *CYP3A4* genotypes *1/*1 or *1/*1B and *1B/*1B were classified as rapid and ultrarapid metabolizers, respectively, and the *CYP3A5* genotypes *1/*1, *1/*3, and *3/*3 were classified as rapid metabolizers, intermediate metabolizers, and poor metabolizers, respectively.

### Randomization and masking

All participants were randomly assigned to receive either 7-day or 14-day vonoprazan-based bismuth quadruple therapy in a 1:1 allocation ratio using a computer-generated randomized list. However, concealment of the randomization sequence was not performed. The generation of the randomization sequence and the allocation of patients to each intervention arm were conducted by a research assistant.

### Statistical analysis

In a previous study, the expected eradication rate of bismuth-containing quadruple therapy for a 7-day regimen was 72%, and that for a 14-day regimen was 96%^14^. Assuming a power of 90%, an alpha of 0.05 (two-sided) and a follow-up loss of 10%, a sample size of 100 patients (50 patients in each group) was determined for this study.

The demographic characteristics, eradication rates, and frequency of adverse events with normal or nonnormal variation were determined using Student’s t test or the chi-square test, as appropriate. The *H. pylori* eradication rates were determined by intention-to-treat and per-protocol analyses for each arm. Statistical significance was defined as a p value < 0.05. All analyses were computed using SPSS version 28.0 (IBM, Armonk, NY, USA).

## Results

### Baseline characteristics

Between June 1, 2021 and March 31, 2022, a total of 337 dyspeptic patients who underwent EGD were assessed for eligibility. Of these patients, 105 (31.1%) were found to have *H. pylori* infection. Four patients were excluded due to incomplete information. A total of 101 patients with *H. pylori* infection were enrolled and randomized to treatment regimens (51 patients receiving 7 days of vonoprazan-based bismuth quadruple therapy and 50 patients receiving 14 days of vonoprazan-based bismuth quadruple therapy). The mean age was 53.4 years, and 53 (52.5%) patients were male. There were no significant differences between the two groups in terms of sex, underlying disease, history of smoking, or alcohol consumption. None of the patients dropped out during the study period. The baseline demographic data are shown in Table 1 and Supplementary Table S1.

**Table 1:** Patient baseline characteristics.

	7-day regimen (n = 51)	14-day regimen (n = 50)	p value
Age, years (mean ± SD)	51.49 ± 12.2	55.36 ± 12.5	0.119
Male, n (%)	23 (45.1%)	30 (60%)	0.165
Underlying disease, n (%)			
Diabetes	7 (13.7%)	5 (10%)	0.760
Hypertension	15 (29.4%)	17 (34%)	0.672
Dyslipidemia	11 (21.6%)	11 (22%)	1.000
Smoker, n (%)	9 (17.6%)	8 (16%)	1.000
Alcohol drinker, n (%)	11 (21.6%)	14 (28%)	0.496

*SD* standard deviation.

**Figure 2:**
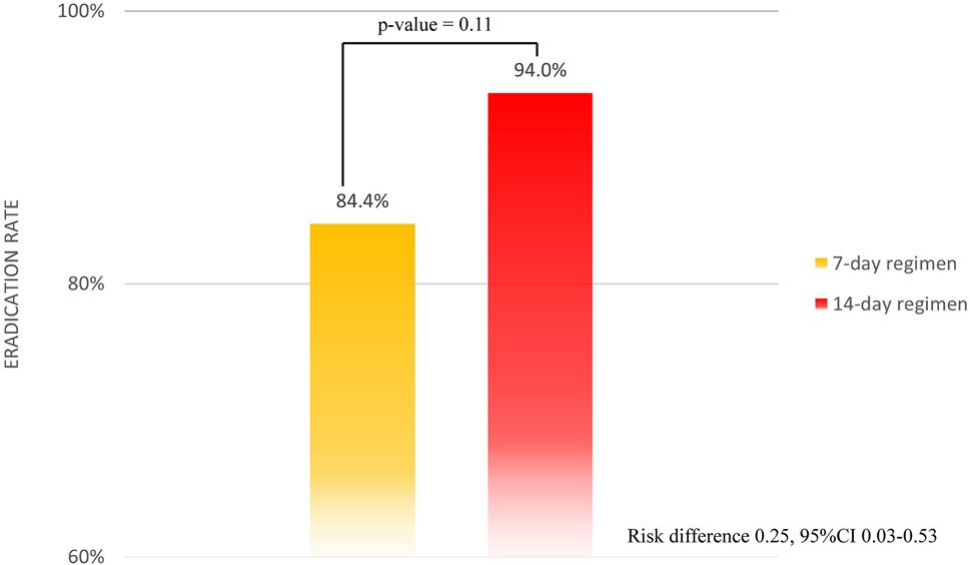
Eradication rates according to the treatment regimen.

The *H. pylori* eradication rates in the 7-day regimen and 14-day vonoprazan-based bismuth quadruple therapy group were 84.4%, 43/51 (95% CI 74.3–94.2) and 94% (47/50; 95% CI 87.4–100), respectively, with a risk difference of 0.25 (95% CI 0.03–0.53, p value = 0.11), as shown in Fig. 2. Both the intention-to-treat and per-protocol analysis results were indistinguishable since there was no dropout during our study protocol.

Antibiotic susceptibility data were available for 18 patients (17 from the E-test and 1 from GenoType® HelicoDR). The antibiotic resistance rates were 33.3% for clarithromycin (6/18), 29.4% for metronidazole (5/18) and 27.7% for levofloxacin (5/18). None of the isolates developed amoxicillin resistance. Interestingly, 14 days of vonoprazan-based bismuth quadruple therapy provided 100% eradication in the clarithromycin-resistant group. However, among the patients in the 7-day regimen group, only two exhibited resistance to clarithromycin. Unfortunately, neither of them achieved a cure from *H. pylori* infection.

In addition, out of the 18 patients with a positive culture for *H. pylori* who experienced treatment failure (2 patients in the 7-day regimen and 4 patients in the 14-day regimen), only one patient exhibited pansensitivity to antibiotics (5.56%). Among the 18 patients, 2 were resistant to levofloxacin (11.11%), 2 were resistant to clarithromycin (11.11%), 1 was resistant to metronidazole (5.56%), and 2 exhibited dual resistance (11.11%).

Regarding the *CYP3A4* and *CYP3A5* genetic polymorphisms, the CYP3A4 genotype accounted for 100% of the rapid metabolizers, and the *CYP3A5* genotype accounted for 7.7%, 72.3%, and 20% of the poor, intermediate and rapid metabolizers, respectively. The *CYP3A4* and *CYP3A5* genotypes and eradication rates are shown in Table 2.

**Table 2:** *CY3A4* and *CYP3A5* genotypes and eradication rates.

	7-day regimen (n = 32)	14-day regimen (n = 33)	p value
CYP3A4 (n = 65)			
RM (n = 65)	27/32 (84.3%)	30/33 (90.9%)	0.475
CYP3A5 (n = 65)			
PM (n = 5)	2/2 (100%)	3/3 (100%)	1.000
IM + RM (n = 60)	25/30 (83.3%)	27/30 (90%)	0.706

*PM* poor metabolizer, *IM* intermediate metabolizer, *RM* rapid metabolizer.

The incidence of adverse events was comparable in both treatment groups, with adverse events occurring in 29.4% (15/51) and 28% (14/50) of patients in the 7-day and 14-day regimens, respectively. The most commonly reported adverse event in both groups was diarrhea (15.7% vs. 12%). All adverse events resolved spontaneously, without the need for intervention or interruption of treatment, and subsequently disappeared after the completion of treatment. No serious adverse events were reported during the study period. Details on adverse events are presented in Table 3.

**Table 3:** Adverse events according to the treatment regimen.

	7-day regimen (n = 51)	14-day regimen (n = 50)	*p* value
Bitter taste, n (%)	4 (7.8%)	8 (16%)	0.205
Diarrhea, n (%)	8 (15.7%)	6 (12%)	0.592
Nausea and vomiting, n (%)	2 (3.9%)	6 (12%)	0.160
Rash, n (%)	1 (2%)	2 (4%)	0.546

## Discussion

*H. pylori* is a spiral-shaped, microaerophilic, and pathogenic gram-negative bacterium that colonizes the stomach. *H. pylori* is present in more than half of the global population, making *H. pylori* infection one of the most common infectious diseases worldwide^15^. Moreover, *H. pylori* is known to cause various gastroduodenal diseases, including chronic gastritis, peptic ulcer disease, mucosa-associated lymphoid tissue lymphoma (MALT lymphoma), and gastric cancer^1–3^. Due to emerging trends of increasing antibiotic-resistant *H. pylori*, eradication with conventional triple therapy has been described to be less effective in several countries, including Thailand^4,7,16^. Successful eradication of *H. pylori* relies on multiple factors, including antibiotic resistance, the suppression of gastric acid, and compliance with treatment. A 2013 national survey in Thailand revealed that at least one antibiotic-resistant strain of *H. pylori* was present in 50.3% of cases, with 36% being metronidazole resistant and with clarithromycin resistance rates ranging from 3.7 to 14%. In regions with high rates of clarithromycin and metronidazole resistance, conventional triple therapy or sequential therapy for *H. pylori* eradication has been deemed ineffective, with eradication rates falling below 80%. Vonoprazan is a potassium-competitive acid blocker (P-CAB) with stronger acid suppression than proton pump inhibitors. Recent studies have reported that the vonoprazan-based regimen has excellent efficacy for *H. pylori* eradication^8,10,13^. Furthermore, a prior study reported that the *H. pylori* eradication rate was more than 90% with bismuth-containing quadruple therapy, irrespective of clarithromycin, levofloxacin or metronidazole resistance^17^. Therefore, the combination of bismuth-containing quadruple therapy and vonoprazan is highly effective for *H. pylori* eradication. These findings are consistent with the Maastricht/Florence VI consensus report stating that P-CAB-based regimens are superior, or not inferior, to conventional PPI-based triple therapy and are superior in patients with antibiotic resistance^7^.

Our previous study of vonoprazan-based regimens showed that both 14-day vonoprazan-based dual therapy and 14-day vonoprazan-based triple therapy had inadequate eradication rates^18^. Our study hypothesized that adding bismuth subsalicylate as a quadruple therapy could achieve a greater eradication rate. Clarithromycin resistance was detected in 33.3% of the strains obtained from gastric mucosal biopsies of the participants who underwent culture testing. This finding is consistent with recent studies that reported clarithromycin resistance rates of 17–43% in Southeast Asia^19,20^. Our study demonstrated that 7 days of vonoprazan-based bismuth quadruple therapy was not effective against clarithromycin-resistant strains, which is consistent with the findings of a study from Korea^21^. However, 14 days of vonoprazan-based bismuth quadruple therapy achieved excellent efficacy against clarithromycin-resistant strains. Therefore, the 14-day regimen may overcome the effect of antimicrobial resistance and could be considered a first-line treatment for *H. pylori* eradication, especially in areas with high levels of clarithromycin resistance. Furthermore, our regimens were well tolerated, and no serious adverse events occurred. Predictable side effects included black stools, bitter taste, and diarrhea, which spontaneously resolved after treatment completion. In a previous study, variations in the eradication rates of vonoprazan-based regimens were reported based on *CYP3A5* genotype^22,23^. Our study showed that our participants were mainly *CYP3A5* intermediate metabolizers, while a study from Japan reported that the majority of their cohort were *CYP3A5* poor metabolizers^22^. Eradication rates were slightly lower in patients who were *CYP3A5* rapid metabolizers than in those who were poor or intermediate metabolizers in both the 7-day and 14-day regimens, consistent with our prior pilot study^18^.

Our study had limitations because it was a single-center study and an open-label clinical trial; hence, bias was inevitable. These findings should be substantiated with further multicenter double-blind trials. Additionally, it would be beneficial for future studies to include a 10-day quadruple treatment group, as it remains uncertain whether this duration could have been sufficient. Furthermore, incorporating a comparator group receiving proton pump inhibitor therapy could provide valuable comparative information for further studies. Despite these limitations, our study had several strengths. First, it was a pioneer randomized controlled trial of vonoprazan-based bismuth quadruple therapy as a first-line therapy for *H. pylori* eradication. Second, this study was conducted in an area with a clarithromycin resistance rate of more than 15%. Both regimens were effective in terms of eradication rates. Moreover, the 14-day regimen achieved excellent eradication among the clarithromycin-resistant strains. Third, no patients dropped out of this study. Moreover, our study demonstrated that the CYP3A4/5 polymorphism potentially influenced the eradication rate, and this result differed from those of other studies.

## Conclusion

In conclusion, this study contributes new evidence by demonstrating the effectiveness of 7-day and 14-day vonoprazan-based bismuth quadruple therapy for *H. pylori* eradication, regardless of clarithromycin or levofloxacin resistance. This study also included a *CYP3A4/5* genotype analysis, which showed that 14 days of vonoprazan-based bismuth quadruple therapy had excellent efficacy for *H. pylori* eradication even in *CYP3A4/5* rapid metabolizers. Therefore, this 14-day regimen can be used as an alternative first-line treatment for *H. pylori* eradication.

### Supplementary Information

Supplementary Table S1.

## Data Availability

The deidentified datasets collected and analyzed during this study are available from the corresponding authors upon reasonable request.
