# Supplementary material for: Fourteen-day vonoprazan-based bismuth quadruple therapy for H. pylori eradication in an area with high clarithromycin and levofloxacin resistance: a prospective randomized study (VQ-HP trial)
Source: Sci Rep. 2024 Apr 18;14:8986. doi: 10.1038/s41598-024-59621-3 (PMC11026498; doi:10.1038/s41598-024-59621-3)
Supplement: Supplementary file 1 — Supplementary Table S1. [file 41598_2024_59621_MOESM1_ESM.pdf]

Table S1: Factors Associated with Successful Eradication in a Multivariate Analysis

Variables	Univariate analysis			Multivariate analysis		
	OR	(95% CI)	p-value	OR	(95% CI)	p-value
Male gender	1.37	(0.39 – 4.82)	0.62	1.01	(0.26 – 4.56)	0.897
Age	1.01	(0.96 – 1.06)	0.67			
Diabetes	5.86	(1.40 - 24.4)	0.02	10.8	(1.71 – 68.0)	0.07
Hypertension	0.19	(0.02 – 1.56)	0.12			
Smoking	0.46	(0.05 – 3.87)	0.48			
Alcohol consumption	0.65	(0.13 – 3.22)	0.59			
